# An endoscopic-assisted contralateral paramedian supracerebellar infratentorial approach in the treatment of thalamic hemorrhage with hematoma extension into the brainstem: a case report

**DOI:** 10.3389/fsurg.2023.1277990

**Published:** 2023-12-19

**Authors:** Eryi Sun, Siyuan Lu, Bo Chen, Qi Wu

**Affiliations:** ^1^ Department of Neurosurgery, Affiliated People's Hospital of Jiangsu University, Zhenjiang, China; ^2^ Department of Radiology, Affiliated People's Hospital of Jiangsu University, Zhenjiang, China

**Keywords:** paramedian supracerebellar infratentorial approach, thalamic hemorrhage with hematoma extension into the brainstem, endoscopic technology, case report, contralateral paramedian supracerebellar infratentorial

## Abstract

**Objective:**

Thalamic hemorrhage (TH) with hematoma extension into the brainstem can lead to poor outcomes. In this study, we discuss the feasibility of the endoscopic-assisted contralateral paramedian supracerebellar infratentorial (SCIT) approach as a therapeutic method for treating such patients.

**Case presentation:**

A patient suffered from a sudden loss of consciousness and right limb weakness, and a CT scan indicated TH with hematoma extension into the brainstem. She consented to undergo surgery by the endoscopic-assisted contralateral paramedian SCIT approach.

**Results:**

Now, the patient can open her eyes on her own and move her left arm in response to commands.

**Conclusion:**

The endoscopic-assisted contralateral paramedian SCIT approach may be a viable therapeutic method for treating TH patients with hematoma extension into the brainstem.

## 1. Introduction

Intracerebral hemorrhage accounts for 6.5%–19.6% of stroke cases (1), and approximately 15% of these cases occur in the thalamus (2). The clinical manifestations and prognosis of thalamic hemorrhage (TH) are related to the size of the hemorrhagic lesion, the direction of the hematoma expansion, and whether it breaches the ventricle. TH with a small and localized hemorrhage usually presents with mild consciousness impairment, typical clinical symptoms, and good prognosis. Conversely, TH with a larger hemorrhage can result in a variety of clinical symptoms and poor prognosis, especially when it is followed by brainstem injury (4). TH and brainstem injuries are both challenging issues of neurosurgery due to their deep location and the complex neural circuitry in the surrounding areas (5). Hemorrhaging in this deep region of the brain involves the same blood vessels as those in the brainstem, causing the spilled blood to expand into adjacent areas and forming a hematoma (3). Currently, there are several surgical therapies available, including stereotaxic intracranial puncture, endoscopic techniques, and microscopic removal of hematoma; however, none of these methods can simultaneously treat hematoma in the thalamus and the brainstem (6, 7). This is attributed to the depth of the hematoma and the obstruction caused by the tentorium (6, 7). In this study, we report a case of a patient with TH with hematoma extension into the brainstem, and discuss the feasibility of using an endoscopic-assisted contralateral paramedian supracerebellar infratentorial (SCIT) approach as a method for treating such patients.

## 2. History

A 64-year-old woman experienced a sudden loss of consciousness and right limb weakness, and she was diagnosed at a local hospital as having experienced a brain hemorrhage. Four hours later, she was transferred to our hospital. She had hypertension and was treated with hypotensive drugs.

## 3. Examination

**Figure 1:**
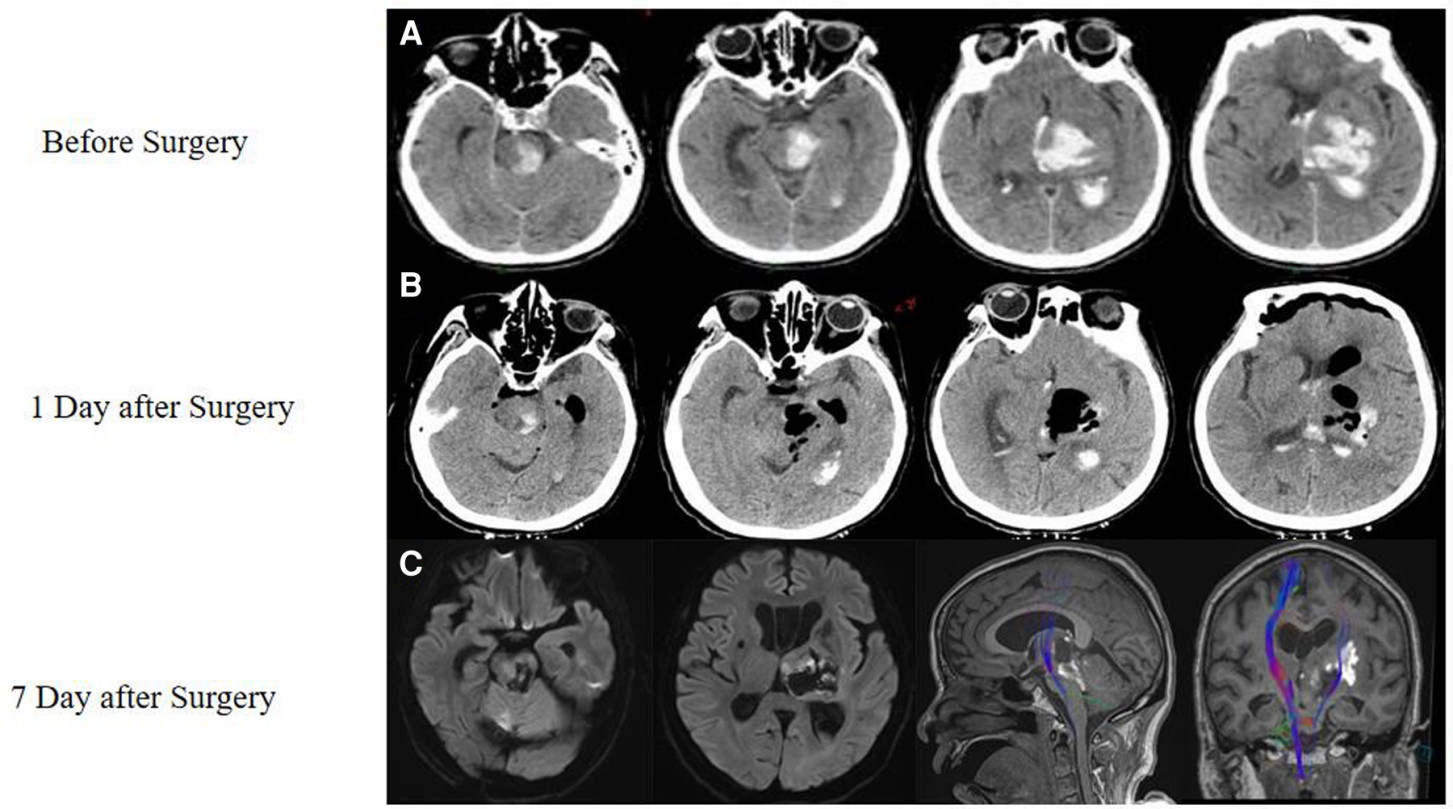
Case of a thalamic hemorrhage with extension to the brainstem. (**A**) An initial CT scan performed in the emergency room showed thalamic hemorrhage with extension to the brainstem and displayed a hematoma from the midbrain to the thalamus. (**B**) On the first day after the surgery, another CT showed that most of the hematoma had been removed. (**C**) Seven days later, a magnetic resonance imaging (MRI) scan was performed, which showed that a part of the white fiber had been saved, as shown by DTI.

A physical examination revealed that the patient was unconscious, unresponsive to pain, and unresponsive to right upper limb stimulation. The Glasgow Coma Scale (GCS) score was 1-1-3. A computed tomography (CT) scan showed TH with hematoma extension into the brainstem (Figure 1A).

## 4. Operation

**Figure 2:**
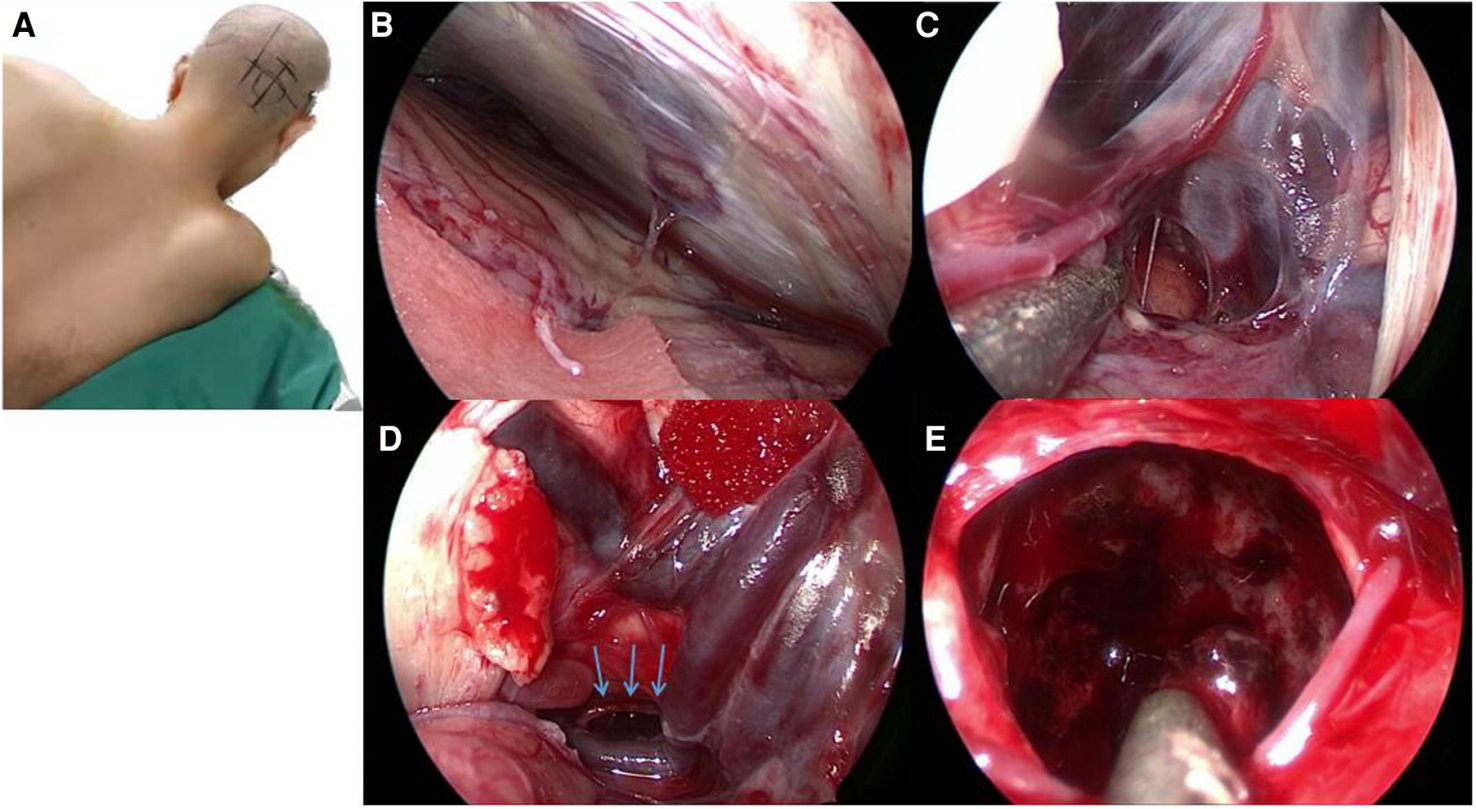
The vision from an endoscope in this case. (**A**) During the operation, the patient was placed in the left lateral oblique position with her upper body elevated by 30°. (**B**) The endoscope was used to access the supracerebellar infratentorial region, and we identified a single bridge vein, which we coagulated with bipolar cautery. (**C**) Gallen vessels and Rosenthal's veins. (**D**) The dorsal part of the pons was also identified, which allowed us to quickly locate and remove the hemorrhage; the blue arrow points to the area of the hematoma. (**E**) We used the endoscope to follow the hemorrhage and removed approximately 40 ml of blood.

During the surgery, the patient was positioned in the left lateral oblique position with her upper body elevated by 30° (Figure 2A). An endoscope monitor (Karl Storz, Germany) and a neuronavigator were placed in front of the patient, while an endoscopic pneumatic holder (Karl Storz, Germany) was positioned on the contralateral bedside to enable one-handed adjustment.

An incision was made 4 cm beside the midline, with a length of 5 cm across the transverse sinus (2 cm above and 3 cm below the transverse sinus). A hole was drilled below the transverse sinus, and a 3 cm × 4 cm bone window was created across the transverse sinus. The semilunar dural flap was opened based on the transverse sinus.

Using the endoscope, we accessed the supracerebellar infratentorial region and identified a single bridge vein, which we coagulated using a bipolar cautery (Figure 2B). We then located the vein of Galen (Figure 2C) and Rosenthal's veins, behind which we found the hemorrhage. The dorsal part of the pons was also identified, enabling us to quickly locate and remove the hemorrhage (Figure 2D). Approximately 40 ml of blood was removed in the process of following the hemorrhage using the endoscope (Figure 2E). The surgery was then concluded.

## 5. Post-operation

One day after the surgery, a CT scan showed that the hemorrhaging had stopped and most of the hematoma had been removed (Figure 1B). Seven days later, a diffusion tensor imaging (DTI) scan showed that some of the white matter structure had been preserved (Figure 1C). At the time, the patient was able to open her eyes independently and moved her left arm in response to commands. Thirty days after surgery, her GCS score improved to 4-T-6.

## 6. Discussion

TH with hematoma extension into the brainstem is associated with a poorer prognosis compared with a localized hemorrhage without vertical extension (4). Extensive hemorrhage in the thalamus can involve adjacent structures such as the brainstem and cerebellum, resulting in primary and secondary damage. The mortality rate of severe TH ranges from 47.0% to 90.0% (3), which is higher than that of cerebral hemorrhage (8). Therefore, it is imperative to develop new treatment methods for treating severe TH.

The methods of treatment for intracerebral hemorrhage include medication and surgery (7); however, medication has limited effect. Surgery has become an important method for treating intracerebral hemorrhage. It can rapidly clear the hematoma, relieve high intracranial pressure, and alleviate mechanical compression. There are several surgical options, including large or small bone window craniotomy and minimally invasive hematoma removal by hematoma puncture and drainage with or without neuroendoscopy (6). However, the management of medial thalamic or brainstem hematomas remains a challenge, especially when the hematoma extends from the thalamus to the brainstem. There are two difficulties in performing surgery on patients with this condition. First, the location of the hematoma is deep, and consequently, for performing a conventional surgery, surgeons may need to sacrifice a part of the normal brain structures to improve visualization, which could result in additional risks. Second, when the thalamic hematoma extends vertically into the brainstem, it requires traversing the tentorium, and current surgical options can help manage only a single hematoma either above or below the tentorium. Therefore, based on the current state of technology, surgery is not recommended for these patients (10).

However, for these patients, we believe that other surgical approaches are crucial. We explored a surgical approach that allows simultaneous access to the posterior, medial, or lateral regions of the thalamus, as well as the brainstem. The thalamus was conceptually divided into six different regions based on the locations reachable by various surgical approaches (9), namely, area 1 (front bottom), area 2 (inside), area 3 (outside), area 4 (back top), area 5 (outside back bottom), and area 6 (inside back bottom) (10). In the case of the patient in our study, we considered a surgical approach that can reach areas 2, 4, and 6.

The superior parietal approach can provide access to the posterior hypothalamus and a part of the adjacent brainstem but requires incisions of the superior longitudinal fasciculus and the carpet fibers (11, 12), which interrupt vital functions such as optic radiations and speech pathways. The same issue arises with the transprecuneus approach, which involves interhemispheric (13) access to the posterior upper thalamus, but requires removal of the corpus callosum tongs, medial cingulate gyrus, upper longitudinal fasciculus I, and precuneus (14), which are involved in higher mental functioning, memory, and other neurocognitive functions (15). The posterior interhemispheric transcallosal approach also needs to cut and pull the pressure part of the corpus callosum, which can cause disconnection syndrome (16). The posterior lateral lower part of the thalamus can be accessed through the paramedian supracerebellar transtentorial approach to the medial cerebellum, which can reach areas 4, 5, and 6 of the thalamus. However, it requires incisions of the cingulate gyrus and fornix, leading to cognitive and behavioral changes and memory impairment. The interhemispheric fissure and transverse fissure approaches provide the most reasonable routes to the thalamus, either through the transcallosal approach or through the transcisternal one. However, even the anterior interhemispheric fissure transcallosal approach requires cutting a portion of the corpus callosum to access the lateral ventricle surface located in the thalamus of the lateral ventricle body, making it less desirable (10). The transcisternal pathway provides access to the cisternal surface of the thalamus without incising any neural structures. The SCIT approach allows access to the posterior lower part of the medial thalamus and the dorsal brainstem through a median, paramedian, or lateral approach. The main disadvantages of the SCIT approach are cerebellar retraction and venous infarction (17), which can be avoided by a paramedian or lateral approach. Furthermore, the brainstem contains numerous nerve nuclei and conducting fibers that are easily damaged during surgery, resulting in neurological dysfunctions. For lesions inside the brainstem, it is necessary to choose an approach that can enter the “safe zone” to ensure minimal damage to the brainstem functional areas. There are 13 safe areas, and studies have shown that by operating in these areas, white matter and other related damage to the patient can be minimized (18). In the case of our patient, we considered accessing the lesion from the lateral mesencephalic sulci or lateral pontine safe areas, which could be reached through the supratentorial approach, the extreme lateral supratentorial approach, the retrosigmoid approach, or the retrolabyrinthine approach. Ultimately, the SCIT approach was chosen after considering all the approaches mentioned above, because it can traverse the tentorium to reach both the brainstem and the thalamus, hence preventing excessive damage to the normal brain tissue.

The SCIT approach provides a relatively broad infratentorial space with familiar anatomical structures and locations. However, the wide application of this approach under the microscope is partly restricted by the deep surgical field, limited illumination, and surgeon fatigue (19). The introduction of an endoscope provides increased visibility and illumination of the surgical field (20), offers a wide-angled panoramic view (21), and allows for minimally invasive craniotomies in approaching deep-seated lesions. The endoscopic-assisted SCIT can manage lesions from the entire infratentorial (from the pineal region to the petrous crest) to the supratentorial space (lateral ventricle, medial surface of the temporal lobe) (22, 23). In this study, the contralateral paramedian SCIT approach was used in the contralateral hemorrhage oblique position. First, this approach lent more comfort to the surgeon's upper arm and provided more space for the surgeon for gravity-induced cerebellar subsidence in the sitting position. Simultaneously, it improved patient safety and prevented serious complications such as air embolism (24). Hence, we used an endoscopic-assisted contralateral paramedian SCIT approach to cover the dorsal brainstem and the inner TH with a panoramic view.

In previous studies, the endoscopic-assisted SCIT approach has been described with case reports and small case series (19, 25, 26). It has been used to deal with pineal and posterior third ventricle lesions in the prone position (19, 26) and thalamic tumors (25). However, this is the first time that it is used in intracerebral hemorrhage surgery. We chose the contralateral paramedian SCIT approach because the thalamus and brainstem bleeding sites are connected to each other, and the positioning of the body enables the surgical approach and the hematoma to maintain roughly the same direction during the operation. This is convenient for visual field adjustment under the control of the pneumatic arm and also convenient for the surgeon to use. Compared with the midline SCIT approach, the bridge veins are significantly reduced in the paramedian SCIT approach, with the number reported to be as low as 0 or 1 (19). This resolves the main disadvantage of SCIT, which can easily cause cerebellar bridge vein occlusion or enlarge the hole in the sinus, resulting in disastrous bleeding (25). The arachnoid membrane anatomy of the quadrigeminal cistern is complex. In addition to the outer sheet-like membranes, it contains arachnoid membranous envelopes wrapped around the vein of Galen and its tributaries, and trabecular membranes are connected to these sleeves (19). We avoided these complex anatomies, and because of the brainstem hemorrhage suffered by the patient, we used the safe zone of the brainstem at the lateral pontine region to resect the hemorrhage. During the surgery, the hemorrhage was so visible that we could minimize damage to any other stem functional areas. We also deliberately chose to use the safe zone in the brainstem to minimize damage to the patient's white matter, reduce other related functional damage, and avoid many important veins in the quadrigeminal cistern, which made the surgery easier to perform.

Despite a global decrease in the mortality rate of patients with cerebral hemorrhage, surgical treatment outcomes for thalamic and brainstem hemorrhages have not shown any improvement (7). Previous randomized controlled trials investigating surgical interventions for intracerebral hemorrhage, including the MISTIE III study (minimally invasive surgery with rt-PA for cerebral hemorrhage), have not demonstrated significant benefits (32–34). Therefore, it is crucial to explore more minimally invasive surgical approaches and strive for maximal hematoma removal as future directions in the management of cerebral hemorrhage. Clinical departments have been hesitant to perform surgeries due to the high rates of death and coma without substantial benefit. However, recent studies have shown reduced mortality rates specifically in cases of posterior and lateral TH (27, 28). Comatose states can potentially arise from damage to the midbrain and the caudal diencephalic region within the brainstem, which are integral components of the ascending reticular activating system (ARAS) responsible for maintaining consciousness and awareness (29, 30). Notably, this may occur when brainstem bleeding volume exceeds 6.225 ml (31). Therefore, patients with a brainstem hematoma volume of less than 6.225 ml and minimal disruption to the ARAS may potentially benefit from surgical intervention. In our patient, despite the posterior and lateral TH extending vertically into the dorsal part of the midbrain, the patient maintained sustained consciousness 30 days after surgery. This highlights the potential advantages of surgery even in extreme scenarios.

Meanwhile, similar to hypertension causing hemorrhages through white matter, the fewer the white matter lesions, the better the brain function is preserved (35). In our patient, we made reasonable use of the mechanism by which bleeding extends along the white matter after intracerebral hemorrhage. We selected appropriate brainstem safety points and a reasonable surgical approach. The white matter fibers were removed only from the injured part of the thalamus, and the related hematoma was removed by endoscopy. This approach greatly reduced the damage to the peripheral nerve nuclei and the blood vessels and fully exposed and cleared the bleeding site. Moreover, it did not cause new white matter damage and avoided the deep cerebral venous plexus, which reduced the difficulty of surgery and relieved edema caused by venous disconnection after surgery. Based on these facts, this approach is recommended for treating medial and posterior thalamic lesions with acceptable complications, which may provide the benefit of survival.

There are several limitations to the endoscopic-assisted contralateral paramedian SCIT approach, including the absence of preoperative planning systems and relevant postoperative evaluations. Hence, further studies are needed.

## Data Availability

The raw data supporting the conclusions of this article will be made available by the authors, without undue reservation.
